# Deep Learning-Based Spread-Spectrum FGSM for Underwater Communication

**DOI:** 10.3390/s20216134

**Published:** 2020-10-28

**Authors:** Zeyad A. H. Qasem, Hamada Esmaiel, Haixin Sun, Jie Qi, Junfeng Wang

**Affiliations:** 1 Department of Information and Communication, School of Informatics, Xiamen University, Xiamen 361005, China; zeyadqasem@stu.xmu.edu.cn (Z.A.H.Q.); h.esmaiel@aswu.edu.eg (H.E.); hxsun@xmu.edu.cn (H.S.); 2 Electrical Engineering Department, Faculty of Engineering, Aswan University, Aswan 81542, Egypt; 3 School of Electronic Science and Engineering, Xiamen University, Xiamen 361005, China; 4 Department of Information and Communications Engineering, School of Electrical and Electronic Engineering, Tianjin University of Technology, Tianjin 300384, China; jfwang@tjut.edu.cn

**Keywords:** MIMO, underwater acoustic communications, energy efficiency and spectral efficiency, SM, FGSM, deep learning, spread spectrum

## Abstract

The limitation of the available channel bandwidth and availability of a sustainable energy source for battery feed sensor nodes are the main challenges in the underwater acoustic communication. Unlike terrestrial’s communication, using multi-input multi-output (MIMO) technologies to overcome the bandwidth limitation problem is highly restricted in underwater acoustic communication by high inter-channel interference (ICI) and the channel multipath effect. Recently, the spatial modulation techniques (SMTs) have been presented as an alternative solution to overcome these issues by transmitting more data bits using the spatial index of antennas transmission. This paper proposes a new scheme of SMT called spread-spectrum fully generalized spatial modulation (SS-FGSM) carrying the information bits not only using the constellated data symbols and index of active antennas as in conventional SMTs, but also transmitting the information bits by using the index of predefined spreading codes. Consequently, most of the information bits are transmitted in the index of the transmitter antenna, and the index of spreading codes. In the proposed scheme, only a few information bits are transmitted physically. By this way, consumed power transmission can be reduced, and we can save the energy of underwater nodes, as well as enhancing the channel utilization. To relax the receiver computational complexity, a low complexity deep learning (DL) detector is proposed for the SS-FGSM scheme as the first attempt in the underwater SMTs-based communication. The simulation results show that the proposed deep learning detector-based SS-FGSM (DLSS-FGSM), compared to the conventional SMTs, can significantly improve the system data rate, average bit error rate, energy efficiency, and receiver’s computational complexity.

## 1. Introduction

Underwater acoustic communication (UWAC) has become one of the critical research fields due to its proliferation of new scientific, industrial, and naval applications [1,2,3]. However, the limitation of the available bandwidth, poor physical link quality, availability of a sustainable energy source for the battery feed sensor nodes, high latency, and attenuation of the acoustic waves by water still represent the main drawbacks of the wireless communication in the ocean’s environment [3,4,5]. Although optical communication, electromagnetic communication, and magnetic induction communication can be employed for the oceanic environment, acoustic communication is considered to be the widest in use since it achieves long communication distances of tens of kilometers for the low data transmission rate and several tens of meters for the high data transmission rate [6,7]. Therefore, multiple-input multiple-output (MIMO) technologies have been introduced as a communication key technology offering an increased capacity and diversity gain of UWAC [8]. However, decoding complexity, power constraint and inter-channel interference (ICI) are the main problems that limit the practical implementation of MIMO technologies in underwater communication [9,10]. A combination between MIMO technologies and code division multiple access (CDMA) was introduced in [11] to avoid the ICI and improve the UWA communication’s spectral efficiency, but in conventional MIMO systems, the power and decoding complexity leads to a small number of radio-frequency chains compared to the available transmit antennas. These problems are more severe in rich underwater multipath channels due to the high cochannel interference problem [11]. Switching diversity and spatial modulation (SM) has been proposed as an attractive solution for exploiting the inherent antenna redundancy problem [12]. The classical switching diversity approach is suitable for known channel cases at the transmitter side, so antenna switching is used to improve the system performance by selecting the antennas corresponding to the best channels and create diversity switching [9]. In SM, additional information is transmitted by the active antenna index instead of creating diversity [13,14].

Recently, space modulation techniques (SMTs) have been developed in academia. Unlike conventional modulation techniques, SMTs use new methods to obtain a high data rate. Therefore, SMTs have been developed as one of the hopeful solutions of the next fifth-generation (5G) wireless mobile networks due to its benefits in terms of energy efficiency (EE), spectrum efficiency (SE), and low system complexity. SM is considered as the main SMT where one or more antennas can be selected for information bits transmission, and the index of the transmitter antennas is utilized as an information-bearing unit to provide higher data rate compared with the conventional single-input-multiple-output (SIMO) systems and avoid the MIMO conventional problems, such as ICI, high decoding complexity, and inter-antennas synchronization. Researchers provided different SMTs schemes to improve the performance of SM [13,14] by emitting the constellated data symbols using multiples antenna [15,16,17], or separating the real and imaginary parts of the constellated data symbol and then transmitting them via one or more transmit antennas [18,19,20]. Some others tried inserting additional dimensions represented by another type of signal constellation to be used as an index and information-bearing [3,12,21].

Orthogonal channels support multiple users into the same time and frequency by using orthogonal codes in direct-sequence code division multiple access (DS-CDMA) scheme. In order to achieve better performance, coded-aided direct sequence spread spectrum (DSSS) [22] and practical low-rate turbo-Hadamard coding techniques [23] were proposed. Motivated by those characteristics, the spread spectrum (SS) was combined with other techniques to achieve a high data rate as in [24,25]. Coded-index modulation (CIM) and generalized coded-index modulation techniques (GCIM) [24,25] has been introduced as a competitive scheme for SMTs. In CIM and GCIM, a predefined spreading codes index has been used to convey additional information instead of transmitting antennas index which used in conventional SMTs. The main advantage of the CIM and GCIM is avoiding the hardware complexity by using single input single output system (SISO) in state of the MIMO system.

Recently, generalized spatial modulation (GSM) and fully generalized spatial modulation (FGSM) [15,16,17] have been proposed to increase the SM achievable data rate. In GSM, to increase the combined number of transmitter antennas and modulation, the constellated information bits are transmitted through more than one antenna to increase the achievable data rate. FGSM combines the ideas of SM and GSM for a high data rate, where one or more antennas are activated to transmit the information bits. In other words, FGSM uses a variable number of transmit antennas as an additional index to achieve a high data rate. FGSM has the advantage of activating fewer transmit antennas compared to GSM to achieve the same data rate and save the consumed power. Quadrature spatial modulation (QSM) [18] and fully quadrature spatial modulation (FQSM) [20] have been presented to transmit the real and imaginary part of the constellated signal through the same/different transmit antennas to achieve high data rate. In QSM and FQSM, the real part of the constellated data symbol is transmitted through one or more transmit antennas and at the same time, the imaginary part is also transmitted through one or more transmit antennas. The different number of transmit antennas in FQSM transmit real and imaginary parts to improve the SE.

Recently, SMTs such as FGSM [17] and enhanced fully generalized spatial modulation (EFGSM) [3] have been proposed to enhance the limited channel bandwidth and reduce the consumed power transmission in the underwater acoustic communication. In the same line, this paper proposes a spread-spectrum fully generalized spatial modulation (SS-FGSM) scheme to increase the available data rate and avoid the high ICI of the conventional MIMO technologies. To relax the system complexity, SS-FGSM with a deep learning (DL) detector (DLSS-FGSM) is proposed in the state of the maximum likelihood (ML) detector. The main contributions of this paper can be summarized as follows:

- The SS-FGSM scheme is proposed, which employs additional index representation by using spreading code to transmit more information data bits. The Walsh code is considered as a spreading code in this paper due to its perfect orthogonality. Thus, SS-FGSM can carry additional bits by index leading to improve both the data rate and the energy efficiency.
- A new detector with low computation complexity is proposed to relax the receiver-ML based detector computation complexity and save the system consumed power. A deep learning-based detector is proposed to be used in the proposed SS-FGSM underwater communication scheme. In order to adapt the deep learning-based detector for the UWA channel, which is considered to be time-varied, data are first preprocessed to recover the UWA channel effects, and then the deep learning is employed for detecting the index of active antennas, spreading codes, and physical transmitted bits jointly.
- The general mathematical framework for the proposed SS-FGSM performances is laid out thoroughly and an extensive simulation is provided to demonstrate the superiority of the proposed scheme over its benchmarks. It is shown that despite the low-complex detector, the proposed scheme is still outperforming the benchmark with the ML.

All the abbreviations used through this paper are mentioned in Table 1, and the rest of this paper is organized as follows: related work is presented in Section 2. Then, the proposed SS-FGSM scheme is presented in Section 3. The analytical analysis of the proposed scheme is presented in Section 4. Finally, the simulation results and the conclusion are presented in Section 5 and Section 6, respectively.

## 2. Related Work

In recent years, many SMTs have been proposed to improve the SE. On one hand, the SMTs can be classified in terms of information carried over the spatial antenna domain to four categories. First, SMTs which activate a few numbers of transmit antennas without transmitting any information bits over these active antennas as in space shift keying modulation (SSK) [26,27,28], generalized space shift keying (GSSK) [29,30] and quadrature space shift keying (QSSK) [18]. Second, transmitting additional information bits over the index of the active antenna in addition to the constellated data symbol over active antennas, such as SM and GSM [13,14], increasing the combination between and FGSM [15,16]. Third, constellated symbols have been split into real and imaginary parts; those real and imaginary parts of constellated symbols are used to increase the combination between transmitting antennas and modulation by mapping them into same/different antennas independently, such as QSM, differential quadrature spatial modulation (DQSM), and generalized quadrature spatial modulation (GQSM) [18,19,20,31,32,33]. Finally, adding a new index into the transmit antennas, such as another constellation signal or predefined spreading codes [12,24,25,34]. On the other hand, the CIM and GCIM have been introduced as a competitive scheme for SMTs to avoid the hardware complexity of the MIMO system using a predefined spreading code index combined with the SISO system.

The SM has been proposed based on the constellation habitual data symbols to be sent through the active antennas out of all transmitting antennas [13,14]. The index of the activated transmit antennas is utilized to carry additional information bits. The achievable data rate of the SM $R_{SM}$ can be expressed as [13,14]:

(1) $$R_{SM}=\;log_{2}\left(M\right)+\;log_{2}\left(N_{t}\right),$$

where M and $N_{t}$ represent the modulation order and the number of transmit antennas, respectively. To improve the SM data rate, the researchers have proposed the GSM [15,16], where the same constellated data is transmitted through more than one transmits antennas to increase the number combination between the active antennas and modulations. Hence, the achieved data rate is improved while the receiver’s complexity is increased compared to SM. The expression of the achievable data rate of GSM $R_{GSM}$ can be represented as [15,16]:

(2) $$R_{GSM}=\;log_{2}\left(M\right)+\;\lfloor log_{2}\left(\begin{array}{c} N_{t}\\ N_{u} \end{array}\right)\rfloor ,$$

where $N_{u}$, ⌊.⌋, and, $\left(\begin{array}{c} .\\ . \end{array}\right)$, represent the number of active antennas out of the transmitting antennas, the floor operator, and the binomial coefficient, respectively. Recently, FGSM [20] has been proposed as a combination of SM and GSM; such combination increases the achievable data rate with different numbers of active antennas during time instant to transmit data constellated symbol. In other words, either one transmits antennas is activated as SM scheme, or multiple transmit antennas are activated as GSM during time instant to transmit the constellated data symbols while the transmit antennas indices are also used as an information-bearing unit. The achievable data rate in FGSM $R_{FGSM}\;$ can be expressed as follows [14,17]:

(3) $$R_{FGSM}=\;log_{2}\left(M\right)+\;\lfloor log_{2}\overset{N_{t}}{\underset{k=1}{\sum }}\left(\begin{array}{c} N_{t}\\ N_{u} \end{array}\right)\rfloor \;=\;log_{2}\left(M\right)+\;N_{t}-1,$$

where k is an indicator for the number of the active antennas at each time slot out of the transmit available antennas. The EFGSM has been proposed as a combination of enhanced SM (ESM) and FGSM. Conveying data bits in the type of signal constellation increase the data bit transmitted over the limited underwater acoustic channel and saves the power consumed in underwater wireless sensor nodes of the internet of underwater things (IoUTs). The EFGSM is capable of achieving data rate as fellows [3]:

(4) $$R_{EFGSM}=\;log_{2}\left(M\right)+\;\lfloor log_{2}\overset{N_{t}}{\underset{k=2}{\sum }}\left(\begin{array}{c} N_{t}\\ N_{u} \end{array}\right)\rfloor +\;log_{2}\left(\frac{M}{2}\right).$$

Based on expanding the constellated data into two orthogonal dimensions; real and imaginary, QSM has been proposed in [18,32,33]. Unlike the SM, GSM, and FGSM, the QSM does not transmit the constellated data including real and imaginary parts via the same transmit antennas, but they are transmitted independently according to the incoming data bits. This way, the achievable data rate increases without increasing the system complexity. The expression of QSM $R_{QSM}$ data rate can be written as [15]:

(5) $$R_{QSM}=\;log_{2}\left(M\right)+\;log_{2}{\left(N_{t}\right)}^{2}.$$

All the above addressing schemes, and others, have been tried to increase the number of combinations between the transmitting antennas and modulations to increase the data sent via the antenna index. Other schemes try to improve the achievable data rate by adding other indices such as signal constellations indices [12,21]. The signal constellation combination with the transmit antennas indices is used to carry additional information by increasing the number of modulations and transmit antenna combinations using primary and secondary constellations.

Despite the advantage of improving the SE using SMTs, competitive studies were presented in [27,28] in order to avoid the requirement of MIMO hardware. In CIM and GCIM [24,25], a predefined spreading code is used to spread the constellated data symbol before transmission, and then use the code index as an information-bearing unit instead of transmitting antenna index in SMTs. In GCIM [28], the real and also imaginary parts of the signal are spread using a predefined code based on the Hadamard–Walsh matrix. Then, at the receiving side, the received signal is multiplied with all possible codes to choose the index of that code whose result is the maximum. Additional data bits are carried by the spreading code index. The achievable data rate by using GCIM $R_{GCIM}$ can be expressed as:

(6) $$R_{GCIM}=\;log_{2}\left(M\right)+\;2\cdot log_{2}\left(N\right),$$

where $\left(N\right)$ is the number of available spreading orthogonal Walsh codes. To improve the performance of SM, spreading codes are used as an additional index into SM with binary phase-shift keying (BPSK) modulation in SM-CIM. In the transmission side, the modulated data was spread using a predefined spread code among the available codes of Hadamard–Walsh matrix and then transmitted through one single antenna out of transmitting antennas. The indices of the spread code and the active antennas are used to carry additional information bits. Consequently, the data rate achieved in spatial modulation coded index modulation (SM-CIM) $R_{SM-CIM}$ is expressed as follows [34]:

(7) $$R_{SM-CIM}=\;2\cdot (log_{2}\left(M\right)+\;log_{2}\left(N\right)).$$

The achievable data rate in (7) is applicable when only binary digital modulation is used, but for higher modulation orders, it becomes impractical.

Generally, the higher transmission data rate in SMTs can be achieved by increasing the bits transmitted via the antenna index to reduce the physical transmitted bits and avoid the ICI with low receiver complexity compared with the conventional MIMO schemes. However, in current SMTs, increasing the transmitted bits requires a high receiver complexity because the ML decoder complexity is increasing exponentially with the transmitted data rate. Therefore, various schemes are aimed to reduce the complexity of the ML decoder [35,36]. Unfortunately, complexity reduction decreases SMTs performance.

Based on those studies mentioned above, a new SS-FGSM scheme is presented to address the significant drawback in the UWA channel bandwidth limitation and the restriction of availability for a sustainable energy sources to feed the battery of the underwater nodes and overcome ICI restriction existed in the conventional MIMO systems. On one hand, implementing the spread spectrum into FGSM to add an additional index as a bear-information unit. On the other hand, proposing a new deep learning-based low complexity receiver for the new scheme avoiding the huge complexity of the ML decoder. This additional index transmits more data bits and enables transmitting few data bits physically and most of the bits are carried through the indices of the active antennas and spreading codes to conserve the energy of the underwater nodes and enhancing the utilization of the underwater channel bandwidth.

## 3. Proposed SS-FGSM Scheme

In this section, details of the proposed SS-FGSM and deep learning detector will be presented. Firstly, the time-domain underwater acoustic channel impulse response is also briefly discussed. Then a review of the conventional FGSM scheme is presented. After that, SS-FGSM system model is explained in detail. Finally, the deep learning-based detector is discussed and presented.

### 3.1. Underwater Acoustic Channel

Assuming an underwater acoustic channel is a linear time-varying (LTV) multipath channel between transmitter 𝒾 and received antenna  j. The impulse response of such underwater channel can be written as [37,38]:

(8) $$h_{𝒾,j;\mu }\left(t;\tau \right)=\;\underset{\mu }{\sum }A_{𝒾,j;\mu }\left(t\right)\delta \left(\tau -\;\tau_{𝒾,j;\mu }\left(t\right)\right).$$

where $A_{𝒾,j;\mu }{\left(t\right)}^{\prime }s$ and $\tau_{𝒾,j;\mu }{\left(t\right)}^{\prime }s$ denote time-varying path amplitudes and delays, respectively. In this channel model, the channel is composed in terms of significant multipath and each path is time-variant in amplitude and delay. Channel tap delay spread $\tau_{max}$ is the difference between the minimum and maximum path delays. In this paper, we assume full knowledge of channel tap delay. As in [37,38], we further assume the following:

1- All channel path delays experience similar Doppler scaling with the data frame transmission time such as:

(9) $$\tau_{𝒾,j;\mu }\left(t\right)=\tau_{𝒾,j;\mu }-\beta_{𝒾,j}t,$$

where $\beta_{𝒾,j}$ is the doppler scaling factor. However, on the other hand when assuming every path has a different Doppler scaling factor, a part of useful signals is treated as additive noise where the overall noise variance can be increased considerably. Moreover, it’s found that as long as the dominant Doppler shift is caused by the direct transmitter/receiver motion, as in the experiment cases. Additionally, the value of the Doppler scale factor is usually less than 0.01 when the relative speed between the transmitter and the receiver is below 10 m/s [37,39]. Therefore this assumption is justified.

2- Constant path amplitude $A_{𝒾,j;\mu }\left(t\right)$ within the data frame transmission time.

3- The underwater channel parameters $A_{𝒾,j;\mu }\left(t\right)$, $\tau_{𝒾,j;\mu }$, $\beta_{𝒾,j}$ are varied slowly during the whole transmission time.

Under these assumptions the underwater acoustic channel can be written as:

(10) $$h_{𝒾,j;\mu }\left(t;\tau \right)=\;\underset{\mu }{\sum }A_{𝒾,j;\mu }\left(t\right)\delta \left(\tau -\;\tau_{𝒾,j;\mu }-\beta_{𝒾,j}t\right).$$

### 3.2. Conventional FGSM Scheme

In this subsection, we briefly review the modulation and demodulation process of the conventional FGSM scheme. In FGSM modulation, the incoming data bits are divided into two subgroups named the physical-bits subgroup and indexed-bits subgroup. The bits of the physical-bits sub-group are modulated using any digital modulation, while the indexed-bits subgroup are transmitted via the index of active antennas based on a mapping table [17]. The FGSM active antennas might be one or more transmitting the same modulated symbol of the physical-bits subgroup. Therefore, the achievable data rate, $R_{FGSM}$ can be explained as in (3). Based on that, if a 4×4  transmit/receive antennas scenario is considered, the achievable data rate of the FGSM will be 5 bits per channel use (bpcu) using quadrature amplitude modulation (QAM) digital modulation. For example, let the following data ${\left[\begin{array}{cc} \underset{\begin{array}{c} physical\\ bits \end{array}}{\underset{︸}{10}} & \underset{\begin{array}{c} indexed\;\\ bits \end{array}}{\underset{︸}{101}} \end{array}\right]}^{T}$ be the incoming data bits to be transmitted. The first two bits are representing the physical bits will be modulated using QAM modulation resulting constellated modulated symbols s, while the three following bits, representing indexed bits, will be transmitted via the index of active antennas based on a mapping table [17]. Based on the mapping table $T_{x1}$ and $T_{x3}$ will be active to transmit the constellated transmitted symbols, s. Therefore, the FGSM $N_{t}\;\times 1$ active antenna vector $x\;\mathrm{will}\;\mathrm{be}\;x=\;{\left[s\;0\;s\;0\right]}^{T}$. Thus, the received FGSM signal on the jth received antennas in MIMO underwater communication system can be represented as follows:

(11) $$y_{j}\left(t\right)=\;\overset{N_{u}}{\underset{q=1}{\sum }}x_{q}\left(t\right)*h_{q,j;\mu }\left(t;\tau \right)+n_{j}\left(t,\tau \right),$$

where $x_{q}\left(t\right)$ denotes the transmitted data symbols from the q active antenna and “∗” denotes the convolution operation, q is the index of active antenna out of available activated transmit antennas $N_{u}$ in each transmission time, and $N_{u}<N_{t}$ in all SMTs and $n_{j}\left(t,\tau \right)$ is the additive white Gaussian noise over the received signal at antenna j with $n\sim CN\left(0,N_{o}\right),$ respectively.

At the receiving end, Maximum likelihood algorithm is employed to estimate x and q, which can be expressed as follows [17]:

(12) $$\left[\begin{array}{cc} \hat{x} & \hat{q} \end{array}\right]=arg\underset{q,\;x}{\min}{\left\|y-\;h_{q}x\right\|}^{2}.$$

### 3.3. SS-FGSM Scheme

Despite the high data rate offered by MIMO communication technologies, MIMO systems suffer from ICI challenges and high receiver computational complexity. MIMO-CDMA has been proposed to overcome the ICI and increase the transmission data rate [11,40]. However, MIMO-CDMA bandwidth is restricted by ICI interference. Unlike conventional MIMO, SMTs represents an alternative solution for data transmission. In SMTs, a part of data bits is encapsulated in the index of transmit antenna. In other words, one or more transmission antennas will be transmitting the same constellated symbol, and their index out of available transmit antennas will be carrying additional information bits avoiding the conventional MIMO ICI by reducing the MIMO order.

**Figure 1:**
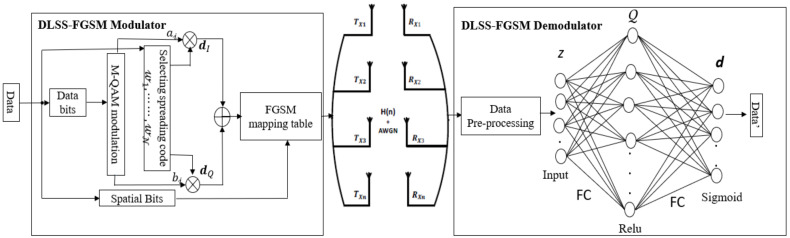
Proposed DLSS-FGSM transmitter and receiver structures.

This paper proposes a new SMTs, Figure 1 shows the proposed system model of the SS-FGSM with low-complexity deep learning receiver (DLSS-FGSM). In the proposed SS-FGSM, the upcoming bit blocks of transmitted data are split into three groups of bits, the first one is the data bits and embeds to $log_{2}\left(M\right)$ bits, the second group is the code index bits and embeds to $2log_{2}\left(N\right)$ bits, and the third group of bits is the antenna index bits and embeds to $\left(N_{t}-1\right)$ bits. The first group is embedded by any digital modulation (e.g., quadrature amplitude modulation (QAM) is used in this paper). Hence, the resulted constellated bits include the real and imaginary parts, $s_{𝒾}=\;a_{𝒾}+jb_{𝒾},$ where $a_{𝒾}$ and $b_{𝒾}$ represent the real and imaginary parts of the constellated symbols in data block 𝒾-th, respectively. Then, $a_{𝒾}$ and $b_{𝒾}$ are spread using the same/different spreading codes according to the incoming bits in the second group of bits. The codes index chosen is used for spreading $a_{𝒾}$ and $b_{𝒾}$ embeds data bits of length $2log_{2}\left(N\right)$. At the end, the spreading signal is transmitted via one or more antennas whose indices carry data bits of length $\left(N_{t}-1\right)$ bits. Therefore, the available data rate in the proposed DLSS-FGSM can be represented as follows:

(13) $$R_{SS-FGSM}=\;\underset{Data\;Bits}{\underset{︸}{log_{2}\left(M\right)}}+\;\underset{Code\;Bits}{\underset{︸}{2log_{2}\left(N\right)}}\;+\underset{Antenna\;Bits}{\underset{︸}{(N_{t}-1).}}$$

As shown in Figure 1, the M-ary modulated symbols and N orthogonal Walsh codes, $W=\left\{{𝓌}_{1},\;\cdots \cdots ,{𝓌}_{N}\right\}$ are employed at the transmitter side. Each spreading orthogonal code consists of L chips with the $T_{c}$ chip period, where 𝓃-th code can be represented in vector form as ${𝓌}_{𝓃}=\left[{𝓌}_{𝓃,1},\cdots \cdots ,{𝓌}_{𝓃,L}\right]{\;}^{T}$.

The transmitted 𝒾-th block of bits in the proposed SS-FGSM can be presented in the vector form as:

(14) $$d_{𝒾}={\left[d_{1,𝒾},\cdots ,d_{\left(2\log_{2}\left(N\right)+\log_{2}\left(M\right)+N_{t}-1\right),𝒾}\right]}^{T}=\;{\left[d_{M-ary,𝒾}^{T}d_{I,𝒾}^{T}\;d_{Q,𝒾}^{T}\;d_{\left(N_{t}-1\right),𝒾}^{T}\;\right]}^{T},$$

where $d_{M-ary,𝒾}^{T}$ is the modulated M-bits subblock. Vectors $d_{I,𝒾}^{T}$ and $d_{Q,𝒾}^{T}$ have $log_{2}\left(N\right)$ bits and it represents the bits transmitted encapsulated in the index of spreading codes for real and imaginary constellation symbols. The signal transmitted of the SS-FGSM scheme can be expressed as:

(15) $$\begin{array}{ll} x_{q}\left(t\right)=\overset{B}{\underset{𝒾=1}{\sum }}\overset{L}{\underset{k=1}{\sum }} & \{a_{𝒾}{𝓌}_{d_{I_{𝓃,𝒾}},k}p\left(t-\left(𝒾L+k\right)T_{c}\right)\;\cos\left(2\pi f_{o}t\right)\\ & \;+\left.b_{𝒾}{𝓌}_{d_{Q_{\overset{´}{𝓃},𝒾}},k}p\left(t-\left(𝒾L+k\right)T_{c}\right)\;\sin\left(2\pi f_{o}t\right)\right\} \end{array}$$

where *B* is the number of transmitted data blocks; ${𝓌}_{d_{I_{𝓃,𝒾}},k}$ and ${𝓌}_{d_{Q_{\overset{´}{𝓃},𝒾}},k}\in 𝓌$ are the consistent spreading sequence for the mapped bits of vector $d_{I,𝒾}^{T}$ and $d_{Q,𝒾}^{T}$, respectively. 𝓃 and $\overset{´}{𝓃}$ are the two indices of selecting spreading codes out of N orthogonal Walsh codes. $p\left(t\right)$ is the pulse shaping filter, in this paper we use a rectangular pulse of unit amplitude in $\left[0,T_{c}\right]$ and $f_{o}$ is the carrier frequency, without loss of generally we use one for time chip $T_{c}$ in this paper.

Table 2 shows an example for the proposed SS-FGSM, assuming a SE needed is 9 bits per channel use (bpcu).

The proposed scheme achieves this SE by only using four transmitter/receiver (Tx/Rx) antennas. For example, consider the incoming block of bits: $d_{𝒾}=\;\left[\begin{array}{ccc} \underset{data}{\underset{︸}{10}} & \underset{code}{\underset{︸}{1011}} & \underset{antenna}{\underset{︸}{111}} \end{array}\right]$, the data modulated bits using any digital modulation (e.g., QAM) are represented in the first two bits $d_{M-ary,𝒾}^{T}=\left[1\;0\right]$. The 1011 four bits represent the code bits, are partitioned into two sub-groups $d_{I,𝒾}^{T}=\left[1\;0\right]$ and $d_{Q,𝒾}^{T}=\left[1\;1\right]$, the first sub-group is used to choose the spreading code used in spreading real constellation symbols $a_{𝒾}$ and the other sub-group is used to choose the spreading code used for spreading the imaginary constellation symbols $b_{𝒾}$. The resulted signal $x_{q}\left(t\right)$ is mapped to be transmitted by one or two antennas based on the next three data bits $d_{\left(N_{t}-1\right),𝒾}^{T}=\left[1\;1\;1\right]$ as in the conventional way of the FGSM scheme [17]. Assuming a full knowledge of underwater acoustic channel matrix H, therefore, the equivalent baseband received signal of the proposed SS-FGSM at the receiver antenna j-th can be expressed as:

(16) $$y_{j}\left(t\right)=\overset{N_{u}}{\underset{q=1}{\sum }}\overset{B}{\underset{𝒾=1}{\sum }}\overset{L}{\underset{k=1}{\sum }}\left\{\begin{array}{c} h_{q,j;\mu }\left(t;\tau \right)a_{𝒾}{𝓌}_{d_{I_{𝓃,𝒾}},k}p\left(t-\left(𝒾L+k\right)T_{c}\right)\;\cos\left(2\pi f_{o}t\right)+\\ h_{q,j;\mu }\left(t;\tau \right)b_{𝒾}{𝓌}_{d_{Q_{\overset{´}{𝓃},𝒾}},k}p\left(t-\left(𝒾L+k\right)T_{c}\right)\;\sin\left(2\pi f_{o}t\right)+{\overset{ˇ}{n}}_{j}\left(t\right)\; \end{array}\right.$$

where ${\overset{ˇ}{n}}_{j}\left(t\right)$ is the term of additive white Gaussian noise after multiplied by the Hadamard–Walsh code.

For the proposed SS-FGSM, the received signal is first pre-processed where the channel is estimated and equalized, and also the other effects such that Doppler effect and carrier frequency offset (CFO) are recovered before detecting the transmitted data. In our proposed scheme, if we use the conventional receiver of SMTs which use the ML decoder to estimate the active antennas indices $\tilde{q}$, spread data symbols $s_{𝒾}$ and the index of the codes used in spreading these symbols, the ML decoder can be expressed in this case as:

(17) $$\left[\tilde{q}\;{\tilde{d}}_{I}\;{\tilde{d}}_{Q}\;{\tilde{s}}_{𝒾}\right]=\arg\underset{q,d_{I},d_{Q},s_{𝒾}}{\underset{︸}{min}}{\left\|y\left(t\right)-\;x_{q}\left(t\right)h\left(t,\tau \right)\right\|}^{2}$$

where $y\left(t\right)={\left[y_{1}^{T},y_{2}^{T},\;\cdots \cdots ,\;y_{N_{r}}^{T}\right]}^{T}$.

The operation of the ML decoder is based on joined the detection of the transmit antenna index, spreading codes index of real and imaginary parts of the modulated signal and the modulated signal itself. Such a process is considered as a high computation detector and the ML decoder complexity is increasing exponentially with all of these estimated parameters. Therefore, a low-complexity detection algorithm is required to relax the complexity of the SS-FGSM.

### 3.4. Deep Learning-Based Detector

In conventional FGSM [17,20], the ML optimal detector is performing a joint detection of the transmit antenna index and transmitted symbol to get the upper bound of the system average BER (ABER). Unfortunately, the ML optimal detector has the highest computational complexity. This paper proposes a deep learning-based detector to relax the computation complexity without high degradation in the BER performance. DL [41] with deep neural networks (DNNs) has been applied to many different fields. Recently, it has been applied to an underwater communication system for the underwater acoustic channel estimation problem [42]. To the best of author’s knowledge, this paper is the first one addressing the DL-based detector in SMTs techniques in general. The DL-based detector is proposed in this paper to reduce the receiver computational complexity, specifically for the FGSM scheme which uses an active antenna number as an additional dimension to carry more information bits. The DL-based detector is proposed as a sub-optimal detector significantly reducing the complexity with acceptable performance. Only two hidden fully-connected (FC) nonlinear layers are used to achieve satisfying performance in the proposed DNN structure. The number of nodes in the hidden layer can adaptively be modified as a trade-off between complexity and performance.

Figure 1 shows the DL-based detector structure. In the receiver side, assuming the channel is fully known, the received signal in (16) is multiplied with $h_{i\forall j}^{-1}\left(t,\tau \right)$ as:

(18) $$r_{i}\left(t\right)=\;h_{i\forall j}^{-1}\left(t,\tau \right)\;y\left(t\right),\;\left(\left(i=1,\;2,\;\cdots ,N_{t}\right)\&\left(j=1,\;2,\;\cdots ,N_{r}\right)\right)$$

where ${\left(.\right)}^{-1}$ is the pseudo-inverse operation. That process will inverse the channel to avoid the effect of the UWA channel. The channel can be estimated and equalized using any other algorithm. Although any other algorithm can be utilized with better performance, in this paper, the Zero forcing (ZF) algorithm is employed for simplicity and better performance is still gained as shown later. The $r_{i}\left(t\right)$, including its real and imaginary parts, is used as the DNN coarse input, where the real part and imaginary part are concatenated and adopted into the input layer.

### 3.5. Structure of DL-Based Detector

The FC nonlinear layers are only required for the proposed DL-based detector, including the first hidden layer with Q nodes and the second hidden layer, corresponds to $d_{𝒾}$ nodes. The output layer is set based on the rate of the proposed SS-FGSM specified in (13). At the hidden layer, the rectifier linear unit (Relu) is utilized as $f_{Relu}\left(x\right)=\;\max\left(x,0\right)\;,$ and the Sigmoid function (Sig) is utilized in the output layer with $f_{Sig}\left(x\right)=$ $\frac{1}{1+\;e^{-x}\;}$ to estimate the transmitted bits $\;{\tilde{d}}_{𝒾}$. Since the output of the sigmoid function lies between 0 and 1, a threshold set to decide either the output is 0 or 1. In this paper we set 0.5 as a threshold and decided the output is 0 if the output less than 0.5 and 1 otherwise. It is worth mentioning that:

(19) $${\tilde{d}}_{𝒾}=\;f_{sig}\left({𝓅}_{2}\;f_{tanh}\left({𝓅}_{1}r_{𝒾}+\;{𝒻}_{1}\right)+\;{𝒻}_{2}\right),$$

where ${𝓅}_{1},\;{𝒻}_{1}$, and ${𝓅}_{2}\;,\;{𝒻}_{2}\;$ is the weights and bias of first and second hidden layers, respectively.

### 3.6. Training Procedure

The DNN model needs to be trained offline then able to use it as a detector. In the training stage, we generate random data sequence and use a measured underwater acoustic channel adopted from an experimental data collection in the ASCOT01 experiment conducted off the coast of New England in June 2001 [17,38] and statistical underwater channel [43], where a different transmitted data frames are generated randomly. The collected received data and channel vectors are pre-process based on Equation (18). By using these collected data, DNN training is processed to minimize the difference between the DNN outputs and the original transmitted data frames. The function loss is defined as follows:

(20) $$f\left(d_{𝒾},\;{\tilde{d}}_{𝒾},\;\theta \right)=\;\frac{1}{R_{DLSS-FGSM}}\;{\left\|d_{𝒾}-{\tilde{d}}_{𝒾}\right\|}^{2},$$

where $\theta =\;\left[𝓅,\;𝒻\right]$ and the θ parameters will be updated for the batches randomly and picked up of the data samples. In our training, the adaptive moment estimation (Adam), which is an advanced updated algorithm based on stochastic gradient descent (SGD), was adopted. Therefore, the parameter θ can be updated as:

(21) $$\theta^{+}:=\theta -\;\alpha \nabla f\left(d_{𝒾},\;{\tilde{d}}_{𝒾},\;\theta \right),$$

where α  indicates the learning rate and ∇ is the gradient operator.

The DL-based detector is very sensitive to the channel signal-to-noise ratio (SNR) $\gamma_{train}$ used so it is selected carefully to let the DL-based detector able for working well at any other SNR. To save the underwater node power, a small value of channel SNR is used in the training stage. Thanks to the orthogonal spreading codes used in the proposed DLSS-FGSM scheme, the proposed DL-based detector can provide good performance if we use $\gamma_{train}=15\;\mathrm{dB}$.

It is worth mentioning that the inputs are concatenated and adopted into the input layer z, that input is representing the pre-processes signal where the channel effects should be estimated and equalized. Additionally, the Doppler effect should be estimated too before adopting the input signal into layer z. The next layer represents the hidden layer, in our case, the hidden layer is represented by  Q. The size of the hidden layer can be adopted as a tradeoff between complexity and performance. Finally, the output layer d has a size of transmitted data rate by blocks including indexed bits carried by the selected spreading code and a physical bit carried by the digital modulation. The size of the output layer d equals the transmitted data rate of each block. It means that no digital demodulation is required in the proposed scheme leading to relaxing more complexity compared to conventional schemes. Hence, the DL detector does not depend on the UWA channel variation; it is only used to detect the received data carried by the indices and digital modulation.

### 3.7. Online Deployment

The online deployment or the deep learning test stage can be performed after the offline training using the optimized θ. Therefore, the received data is pre-processed and put in the DNN to estimate the transmitted bits in real-time. In other words, the proposed DL-based detector does not need extra training for updating the parameter θ. Most importantly, our detector achieves better results with less complexity and without extra training for the DL model.

## 4. SS-FGSM Performance Analysis

In this section, the performance of the proposed SS-FGSM is addressed in terms of ABER performance, energy efficiency, and receiver complexity.

### 4.1. ABER Performance Analysis

In this subsection, we will analyze the ABER performance of the SS-FGSM ML-based detector (MLSS-FGSM). The ABER is a function of the probability of corrupted bits due to detecting the transmit active antennas, spreading codes of real and imaginary parts of the modulated signal, and the modulated signal itself. In other words, ABER is a function of physical transmitted modulated bits $log_{2}\left(M\right)$ and mapped data bits, $2log_{2}\left(N\right)+(N_{t}-1)$ including the bits transmitted by indices of antennas and the bits transmitted by the indices of spreading codes. We denote the probability associated with erroneous antenna detection by $F_{a};$ the probability associated with erroneous spreading code by $F_{c}$; and finally, the probability associated with the modulated bits by $F_{m}$. The ABER of the proposed SS-FGSM scheme can be formulated as:

(22) $$ABER=\frac{\left(N_{t}-1\right)}{R_{DLSS-FGSM}}\;F_{a}+\;\frac{2log_{2}\left(N\right)}{R_{DLSS-FGSM}}F_{c}+\;\frac{log_{2}\left(M\right)}{R_{DLSS-FGSM}}F_{m}.$$

In the MLSS-FGSM, the probability of the errors in the bits transmitted in the index of antenna $F_{a}$ and probability of error of the modulated symbols $F_{m}$ are an independent probability. The probability of the errors in the bits transmitted using the index of the code $F_{c}$ are dependent probability and it depends on the probability of error on estimated index antenna $F_{a}$.

The probability errors in the received bits transmitted using the index of the codes $F_{c}$ can be in two cases: Case 1: if the detection of transmitting active antennas is correct, but the spreading code is detected incorrectly; Case 2: if the detection of the active antennas and spreading codes both are incorrect. Hence, the errors probability $F_{c}$ in the received bits transmitted via the index of the codes can be written as:

(23) $$F_{c}=F_{a}+\frac{\left(1-F_{a}\right)}{N}.$$

and $F_{a}$ can be calculated as [36]:

(24) $$F_{a}\leq \;\frac{1}{2^{\left(N_{t}-1\right)}}\;\overset{2^{\left(N_{t}-1\right)}}{\underset{𝓋=1}{\sum }}\overset{2^{\left(N_{t}-1\right)}}{\underset{𝓀=1}{\sum }}\frac{S\left(q\to \tilde{q}\right)\;{\left(\frac{1}{4\lambda_{a}}\right)}^{N_{r}}\left(\begin{array}{c} 2N_{r}-1\\ N_{r} \end{array}\right)}{\left(N_{t}-1\right)},$$

where $\lambda_{a}=\frac{1}{2}\left(1-\;\sqrt{\frac{\sigma_{a}^{2}}{1+\sigma_{a}^{2}}}\right)$ and $\sigma_{a}^{2}=\;\frac{\gamma }{2}{\left|x_{q}\left(t\right)\right|}^{2}$. $S\left(q\to \tilde{q}\right)$ denotes the number of bits in error between the transmitted bits via the antenna index and its estimation at the receiver side.

The error probability in M-ary modulation can be written as [10]:

(25) $$F_{m}=\;\left(\left(1-\frac{1}{\sqrt{M}}\right)erfc\left(\sqrt{\frac{3E_{s}}{N_{o}\left(M-1\right)}}\right)\right)$$

where $E_{s}\;$ is the energy symbol and $N_{o}$ is the noise variance.

### 4.2. Energy Efficiency 

In the proposed SS-FGSM, $\underset{Data\;Bits}{\underset{︸}{log_{2}\left(M\right)}}$ bits are transmitted physically, but most of transmitting bits $\underset{Code\;Bits}{\underset{︸}{2log_{2}\left(N\right)}}\;+\underset{\;Antenna\;Bits}{\underset{︸}{\left(N_{t}-1\right)}}$ are conveyed in the indices of either transmit active antennas or spreading codes. To calculate the EE of the proposed SS-FGSM scheme, consider each modulated bit requires the energy of $E_{b}$ to be transmitted over the underwater acoustic channel. In SMTs mapping, part of the required transmitted bits is transmitted in the spatial domain to reduce the power consumed in the communication transmission. Therefore, the percentage of energy-saving η of the SMTs compared to the conventional M-ary modulation schemes for the same number of transmitted bits can be calculated as:

(26) $$\eta_{SM}=\;\frac{log_{2}\left(N_{t}\right)}{log_{2}\left(M\right)+log_{2}\left(N_{t}\right)}\%,$$

(27) $$\eta_{GSM}=\;\frac{\left\lfloor log_{2}\left(\begin{array}{c} N_{t}\\ N_{u} \end{array}\right)\right\rfloor }{log_{2}\left(M\right)+\lfloor log_{2}\left(\begin{array}{c} N_{t}\\ N_{u} \end{array}\right)\rfloor }\%,$$

(28) $$\eta_{QSM}=\;\frac{2log_{2}\left(N_{t}\right)}{log_{2}\left(M\right)+2log_{2}\left(N_{t}\right)}\%,$$

(29) $$\eta_{FGSM}=\;\frac{\left(N_{t}-1\right)}{log_{2}\left(M\right)+\left(N_{t}-1\right)}\%,$$

(30) $$\eta_{EFGSM}=\;\frac{\left\lfloor log_{2}\sum_{N_{u}=2}^{N_{t}}\left(\begin{array}{c} N_{t}\\ N_{u} \end{array}\right)\right\rfloor }{log_{2}\left(M\right)+\;log_{2}\left(\frac{M}{2}\right)+\;\lfloor log_{2}\sum_{N_{u}=2}^{N_{t}}\left(\begin{array}{c} N_{t}\\ N_{u} \end{array}\right)\;\rfloor }\%,$$

(31) $$\eta_{GCIM}=\;\frac{2log_{2}\left(N\right)}{log_{2}\left(M\right)+2log_{2}\left(N\right)\;}\%,$$

(32) $$\eta_{SS-FGSM}=\;\frac{2log_{2}\left(N\right)+\left(N_{t}-1\right)}{log_{2}\left(M\right)+2\;log_{2}\left(N\right)+\left(N_{t}-1\right)}\%$$

Clearly, as the number of bits transmitted by the index of the spatial domain of SMTs increases, the EE of the system will be improved and the transmission consumed power will be reduced.

### 4.3. Receiver Complexity

To calculate the receiver complexity, we consider any floating-point number (flops) required in each decision of the receiver decoder. The detection computation complexity at the receiver side is determined by calculating the total number of flops (TNCO) required for each detection method. The current schemes of the SMTs are required to perform searching between a combination of $\;{\left(2\right)}^{R}$, where R denotes the data rate achieved by each SMTs scheme. In addition, we consider in each operation, $\overset{N_{r}}{\underset{j=1}{\sum }}{\left\|y_{j}\left(t\right)-\;x_{q}\left(t\right)h_{qj}\left(t,\tau \right)\right\|}^{2}$ one combined multiplication (i.e., four actual multiplications) to compute $x_{q}\left(t\right)h_{qj}\left(t,\tau \right)$ and four real multiplications to calculate the square norm. The conventional SM has utilized the ML decoder as a detection algorithm and by using the computation of $\overset{N_{r}}{\underset{j=1}{\sum }}{\left\|y_{j}\left(t\right)-\;x_{q}\left(t\right)h_{qj}\left(t,\tau \right)\right\|}^{2}$ as a definition to calculate the TNCO, the SM receiver’s complexity can be expressed as follows [17]:

(33) $${\mathrm{TNCO}}_{SM}=8N_{r}{\left(2\right)}^{R_{SM}}.$$

In the same way, the receiver’s complexity of the GSM has been computed as in the conventional ML detector. However, as multiple transmit antennas are activated in GSM to transmit the same constellated symbol, its receiver’s complexity is increased compared to the conventional SM. The GSM receiver’s complexity is expressed as follows [17]:

(34) $${\mathrm{TNCO}}_{GSM}=8N_{r}\left(2\left(N_{u}-1\right)\right){\left(2\right)}^{R_{GSM}}.$$

The maximum TNCO required for the ML detector in the case of QSM is expressed as follows [17]:

(35) $${\mathrm{TNCO}}_{QSM}=8N_{r}{\left(2\right)}^{R_{QSM}}.$$

In FGSM, the receiver’s complexity is different, where for the number of transmit ML detector has $\frac{N_{t}}{2}-1$ maximum summation for the number of transmitting antennas are more than three, $N_{t}\geq 3$. The complexity computation of the FGSM is expressed as follows [17]:

(36) $${\mathrm{TNCO}}_{FGSM}=8N_{r}\left(2\lceil \frac{N_{t}}{2}-1\rceil \right){\left(2\right)}^{R_{FGSM}}.$$

For the EFGSM, the receiver’s complexity has been decreased significantly since the search of ML is divided into two independent groups represented by primary constellation, carrying $log_{2}\left(M\right)$ bits and secondary constellation carrying $log_{2}\left(\frac{M}{2}\right)$ bits. The EFGSM receiver’s complexity is expressed as follows [20]:

(37) $${\mathrm{TNCO}}_{EFGSM}\;=\;8N_{r}\left[\left(\left(2\lceil \frac{N_{t}}{2}-1\rceil \right){\left(2\right)}^{R_{P}}\right)+\;\left(\left(2\lceil \frac{N_{t}}{2}-1\rceil \right){\left(2\right)}^{R_{S}}\right)\right],$$

where $R_{P}$ is the data bit rate transmitted over the primary signal constellation and it is equal to $log_{2}\left(M\right)$, while the $R_{S}$ is the data bit rate transmitted over the secondary signal constellation and it is equal to $log_{2}\left(\frac{M}{2}\right)$.

In the GCIM, the receiver’s complexity depends on the dispreading of real and imaginary parts of modulated data and the demodulation operation. Hence, in each real and imaginary parts of the received signal, the signal is multiplied first by N codes, each code has N  length before summation to find the maximum value and get the correct spreading code used in mapping data. Then, the dispreads signal is demodulated following the modulation type of transmitter. Therefore, complexity computation of GCIM can be expressed as follows:

(38) $${\mathrm{TNCO}}_{GCIM}=2N^{2}+2N+2^{log_{2}\left(M\right)}.$$

For the proposed scheme, we calculated the computation complexity for two types of detector, ML-detection based, and DL-detection based. For MLSS-FGSM, the complexity computation is calculated as in the conventional FGSM multiplied by complexity due to the estimation of spreading codes. Therefore, the complexity computation of MLSS-FGSM can be expressed as follows:

(39) $${\mathrm{TNCO}}_{MLSS-FGSM}=8NN_{r}\left(2\lceil \frac{N_{t}}{2}-1\rceil \right){\left(2\right)}^{R_{SS-FGSM}},$$

In the case, the SS-FGSM is based on DL-detection, the complexity contribution is computed in different ways since the DL-based detector is used. As shown in (19), the input of the DNN model is first multiplied by the weights of the first hidden layers and summed with the biases of that layer. The resultant signal passed through the activation function, and similarly multiplied and summed with weights and biases of the next layer, respectively. Using that way, the complexity of the DNN model obtained by the training can be calculated by multiplying the output of the input layer with the size of the next layer and then add the biases whose size is similar to the number of nodes of the hidden layer, i.e., the real and imaginary signals of the received data $r_{𝒾}\left(t\right)$ signal is multiplied by the weight of first hidden layer Q. Then every node bias is added, this process is repeated at each hidden layer. Based on that, the DNN complexity can be expressed as:

(40) $${\mathrm{TNCO}}_{DLSS-FGSM}=\;\left(z*\;Q\;+\;Q\right)+\;\left(d*\;Q\;+\;d\right),$$

where z is the number of nodes of the input layer.

## 5. Simulation Results

The performance of the proposed SS-FGSM is evaluated using Monte Carlo simulation over a statistical underwater channel [43] and a measured uncorrelated underwater acoustic channel adopted from an experimental data collection in the ASCOT01 experiment conducted off the coast of New England in June 2001 [17,38]. Both channels are truncated to have an order of μ=128. First, for the statistical underwater channel, the same scenario as shown in Figure 1 was deployed where 4 $T_{x}$ and 4 $R_{x}$ are employed to validate the simulation with a distance of 500 m separating between $T_{x}$ and $R_{x}$. The depth of the $T_{x}$ is covering 20 to 34 m and the same depth for $R_{x}$ with DSF of 4×10^−3^. Both simulation and the measured channels used $f_{o}$ centered at 3550 Hz with channel bandwidth of 0.5 kHz with a frequency range of 3050–4050 Hz. Second, in the measurement channel, the source is deployed 4 m above the bottom and the depth of the bottom is 103 m, 4 $T_{x}$ are employed for our simulation. Additionally, 4 $R_{x}$ are deployed as a vertical vector sensor array covering a depth of 30 to 46 m. The source-receiver range was approximately 1 km. A low frequency modulated (LFM) signal is repeatedly transmitted every 120 s for a period of 160 min. By using a matched filter, the LFM received signal, estimates each channel actuary on a large time scale of 120 s. Using the estimated channel, we compute the equalizer coefficients used for channel equalization. The simulation outcomes are achieved for $10^{6}$ symbols for trusted simulation data due to the added Gaussian noise. In the proposed SS-FGSM based on the DL detector, the training was performed with $10^{3}$ epochs, each contains 20 batches of $10^{3}$ data samples. The learning rate α was set to be $10^{-3}$. The number of nodes in the input layer of DNN was set to double of the length of the received signal since the real and imaginary parts of the received signal were concatenated, the number of nodes in Q was set to be 128 and finally in output layer with a number of nodes equal to the size of incoming data block $d_{𝒾}$. The performance of the proposed scheme is compared with the conventional SMTs, such as SM [9], GSM [12], QSM [15], FGSM [14], GCIM [28], and EFGSM [20], in terms of the achievable data rate, ABER, EE, and receiver’s complexity.

### 5.1. Achievable Data Rate 

The available data rate of the spatial modulation techniques (SMTs) can be dependent on one of two axes, the number of antenna transmitter $N_{t}$ and spreading code length N. Based on these two axes, the SMTs can be classified into three categories; first, SMTs schemes which are dependent only on the number of antenna transmitter $N_{t}$ and convey the transmitted index bits in the spatial domain of the transmitted active antenna as in case of the SM, GSM, QSM, FGSM, and EFGSM. Second, the SMTs dependent only on the spreading code length N and convey the transmitted index bits in the code index as the GCIM which considered as a SISO system. Third, the SMTs dependent on two axes the number of antenna transmitter $N_{t}$ and spreading code length N as in the case of the proposed scheme, and according to our knowledge, it is the first work in this category. Under infinite SNR values, this paper compares the proposed SS-FGSM achievable data rate (13) with the maximum achievable data rate of SM, GSM, FGSM, EFGSM, QSM, and GCIM provided by (1)–(6), respectively. The achievable data rate comparison is considered in three cases. First, only the axis of the number of antenna transmitter $N_{t}$ is varied and the axis of spreading code length N is constant. Second, only the axis of the number of antenna transmitter $N_{t}$ is constant, and the axis of spreading code length N is varied. Third, the axis of the number of antenna transmitter $N_{t}$ and the axis of spreading code length N are varied simultaneously.

All these cases have been addressed for the achievable data rate under the same modulation order, 4-QAM. Furthermore, only two activated antennas out of available transmit antennas used for transmitting the constellated data symbols $N_{u}$ are chosen to avoid the possibility of having the same antenna index in the different antenna subset of GSM. For EFGSM, the primary signal constellation and secondary signal constellation are chosen to be QAM and BPSK, respectively.

(1) Achievable Data Rate at Different Number of $N_{t}$ and Fixed Spreading Codes N.

**Figure 2:**
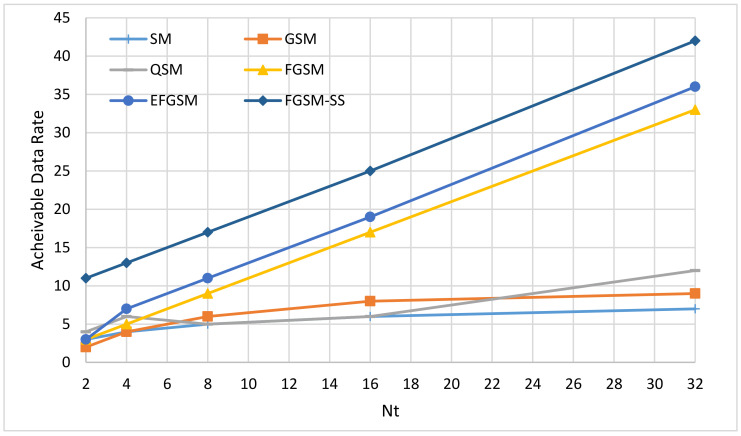
The maximum achievable data rates of different SMTs under different number of $N_{t}$.

The different number of transmitter antenna $N_{t}$ and weighted against the SMTs which are only dependent on the value of $N_{t}$. For the proposed SS-FGSM scheme, the length of the orthogonal code N is fixed to be 16. The results of the comparison are depicted in Figure 2. The proposed SS-FGSM outperforms all conventional schemes in the achievable data rate in this case. For example, at $N_{t}$ = 8, the achievable SE of SS-FGSM is 17 bpcu compared to 5 bpcu, 6 bpcu, 5 bpcu, 9 bpcu, and 11 bpcu of SM, GSM, FGSM, QSM, and EFGSM, respectively.

(2) Achievable Data Rate at Different Number of N and Fixed Number of Transmitted Antennas $N_{t}$.

**Figure 3:**
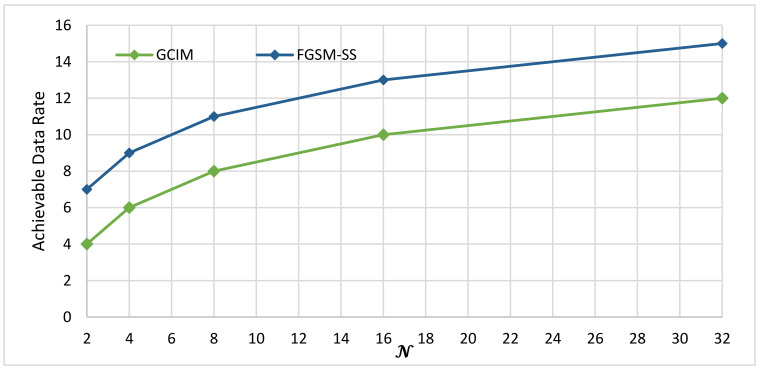
The maximum achievable data rates of GCIM and proposed DLSS-FGSM under different code length N.

As in Equation (5), the achievable data rate of the GCIM is independent on the number of the antenna transmitter $N_{t}$ and it is a function only on the length of the spreading code N to compare the proposed scheme with GCIM. In this case, we fixed the number of transmit antenna to be $N_{t}=4$. The results of this comparison are shown in Figure 3. The proposed DLSS-FGSM outperforms the GCIM scheme in terms of the achievable data rate ($N_{t}-1$). For example, when N = 8, the achievable SE of FGSM-SS is 11 bpcu compared to 8 bpcu for GCIM.

(3) Achievable Data Rate at Different Number of Spreading Codes N and Different Value of Transmitted Antennas $N_{t}$.

**Figure 4:**
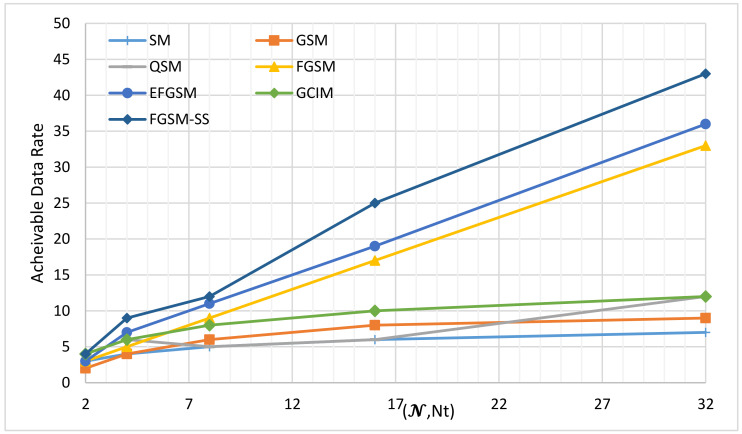
The maximum achievable data rates of different SMTs under different number of $N_{t}$ and code length 𝓝.

This case has a full utilization of the feature of our proposed scheme. Where the proposed scheme uses the two axes of index modulation code and transmitted antenna for simplicity. In this experiment, consider the N and $N_{t}$ are equal. Figure 4, shows the achievable data rate of the different SMTs at the same modulation order, the X-axis in Figure 4 represents the different number of antennas transmitting $N_{t}$ and the length of the spreading code N. The number of antennas transmitting $N_{t}$ will be affected on the SM, GSM, QSM, FGSM, EFGSM, and the proposed SS-FGSM while the different length of the spreading code N will be affected on the GCIM and the proposed SS-FGSM scheme as well. In Figure 4 the achievable data rate of the proposed SS-FGSM is weighted against the maximum achievable data rate of SM, GSM, FGSM, EFGSM, QSM, and GCIM provided by (4)–(8), and (11), respectively. The proposed SS-FGSM is significantly outperformed all of the conventional schemes in the achievable data rate. For example, at $N_{t}=N=4$, the achievable SE of SS-FGSM is 9 bpcu compared to 4, 4, 5, 6, 7, and 6 bpcu of SM, GSM, FGSM, QSM, EFGSM, and GCIM, respectively.

### 5.2. ABER Performance Analysis

**Figure 5:**
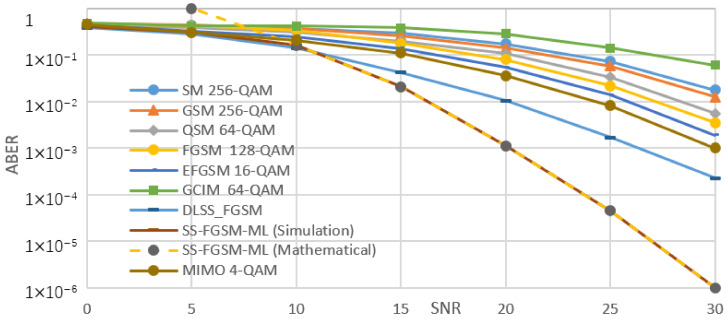
The performance of ABER of proposed SS-FGSM and conventional SMTs at 10 bpcu (over a statistical UWA channel).

**Figure 6:**
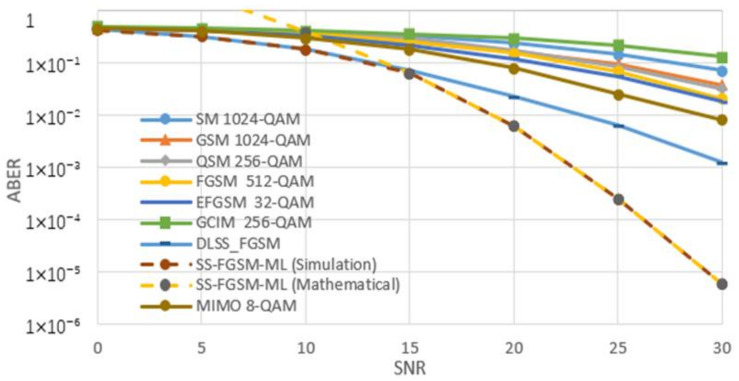
The performance of ABER of proposed SS-FGSM and conventional SMTs at 12 bpcu (over a statistical UWA channel).

**Figure 7:**
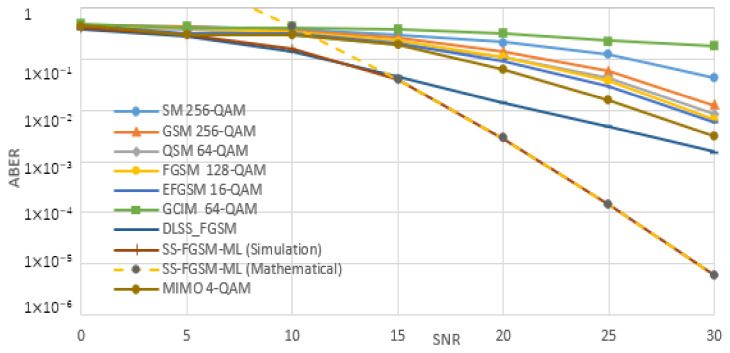
The performance of ABER of proposed SS-FGSM and conventional SMTs at 10 bpcu (measured).

**Figure 8:**
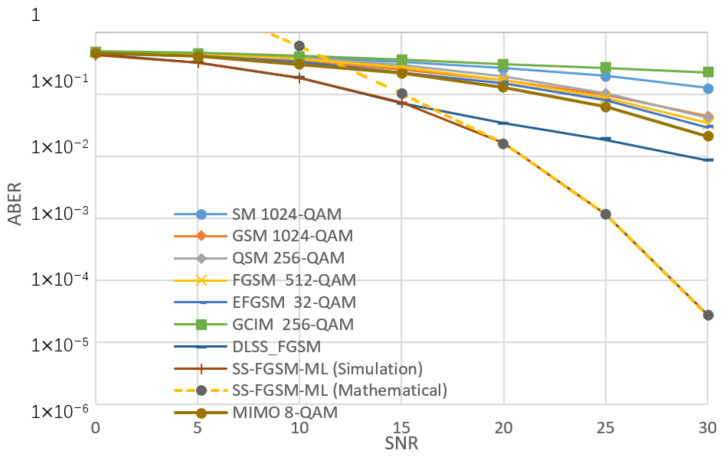
The performance of ABER of proposed SS-FGSM and conventional SMTs at 12 bpcu (measured).

Figure 5, Figure 6, Figure 7 and Figure 8, shows the ABER performance of the proposed SS-FGSM compared with the ABER of conventional MIMO and SMTs. Figure 5 and Figure 6 show the performance of average bit error rate (ABER) over the statistical simulation channel and Figure 7 and Figure 8 show the performance over the measured channel. In Figure 5 and Figure 7 the conventional MIMO achieves 8 bpcu since the achieved data rate in MIMO must be multiple of the number of transmitting antennas $N_{t}$. SM, GSM, QSM, FGSM, EFGSM, MIMO, and SS-FGSM, all schemes have a transmitted and receive antenna configuration of 4×4 MIMO system, while GCIM has SISO transmission system and the length of the spreading code of the proposed SS-FGSM and GCIM are N=4. To achieve 10 bpcu SE in the SMTs with these spreading code length and antenna configuration, the QAM modulation orders are 256, 256, 64, 128, 64, 4, and 8 in SM, GSM, QSM, FGSM, GCIM, and conventional MIMO and the proposed SS-FGSM, respectively. The EFGSM will have a 16-QAM as a primary constellation and 8-PSK as a secondary signal constellation. On the other hand, to achieve 12 bpcu SE, the modulation orders are 1024, 1024, 256, 512, 256, 8, and 32 in SM, GSM, QSM, FGSM, GCIM, conventional MIMO and proposed SS-FGSM, respectively. In the case of 12 bpcu, the EFGSM will use a 32-QAM as a primary constellation and 16-PSK as a secondary signal constellation. As shown, the proposed scheme offers up to 5 dB to 6 dB SNR improvement over all of the conventional SMTs schemes. That manifests an essential remark that in the SS-FGSM scheme, the Euclidean distance between the transmitted data is larger than that in the conventional schemes, also due to noise reduction characteristics of the orthogonal spreading codes used in the proposed SS-FGSM scheme. Although, these spreading codes used in the GCIM, it has a poor ABER performance in underwater communication. Because of the limited achievable data rate of GCIM, the modulation order should be increased to have the same spectral efficiency as in the proposed SS-FGSM. To avoid that, the GCIM can use long orthogonal codes with less mapped bits to provide a better performance, but unfortunately, that increases the system complexity also. The proposed SS-FGSM based on ML-detector provide high ABER performance compare to the SS-FGSM using a DL-based detector, but unfortunately, the ML-detector based has high competition complexity. In addition, it’s shown that the upper bound and the results of asymptotic ABER in Equation (26) of proposed SS-FGSM using ML-detector are matched to the simulation result especially for a wide range of SNR values.

### 5.3. Energy Efficiency Analysis

**Figure 9:**
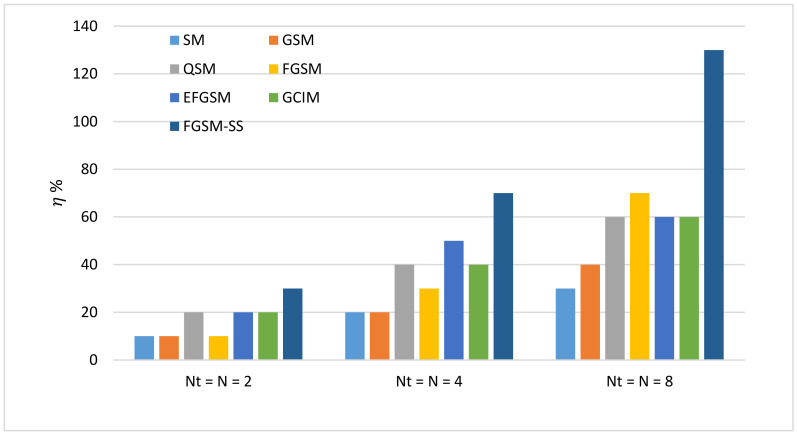
Performance comparison of energy efficiency.

In order to calculate the EE of the proposed scheme, if the underwater node requires to transmit data over the underwater channel with a data rate of 10 bpcu. Unlike the conventional M-ary signal constellation which achieves the rate by modulation order of 1024, the SMTs convey the information bits in the signal constellation, antenna index, and index of spreading codes. Hence, the SMTs physically transmit only the M-ary signal constellation. By this concept, as discussed in Section 4, we can calculate the energy saving achieved by mapping part of the transmitted bits into antennas index or spreading codes. The energy-saving percentage of SM, GSM, QSM, FGSM, EFGSM, GCIM, and SS-FGSM methods are given by (30)–(36), respectively. Figure 9, shows the energy-saving percentage at different number of the antenna transmit $N_{t}$ and different spreading code 𝒩. When $N_{t}=N=2$, the proposed DLSS-FGSM scheme achieves energy saving with a percentage of 30% compared to 10%, 10%, 20%, 10%, 20%, and 20% for SM, GSM, QSM, FGSM, EFGSM, and GCIM, respectively. In case of $N_{t}=N=4$, the proposed SS-FGSM can achieve energy saving with a percentage of 70% compare to 20%, 20%, 40%, 30%, 50%, and 40% for SM, GSM, QSM, FGSM, EFGSM, and GCIM, respectively. In the case of $N_{t}=N=8$, the proposed SS-FGSM can achieve energy saving with a percentage of 130% means that 13 bpcu compared to 30%, 40%, 60%, 70%, 60%, and 60% for SM, GSM, QSM, FGSM, EFGSM, and GCIM, respectively. This high performance of the proposed scheme in EE is due to a low number of bits transmit physically, where it uses two axes of the spatial domain to convey additional information bits while other SMTs use only one axis for index information.

### 5.4. Receiver’s Complexity

In this part, computation complexity (i.e., TNRO) of the decoders used in the proposed SS-FGSM is evaluated and compared to the ML decoders computation complexity used in the SM, GSM, QSM, FGSM, EFGSM, and GCIM. To analyze the computational complexity of SMTs studied in this paper, we assume all schemes are evaluated under the same SE (e.g., 10 bpcu), with antenna configuration of 4×4 MIMO system for SM, GSM, QSM, FGSM, EFGSM, and the proposed SS-FGSM and SISO antenna configuration for GCIM scheme. We also assume the same spreading code length for GCIM and SS-FGSM schemes. The comparison is evaluated under an M-QAM modulation order of 256, 256, 64, 128, 16, 64, and 8 for the SM, GSM, QSM, FGSM, EFGSM, GCIM, MLSS-FGSM, and DLSS-FGSM, respectively. The computation complexity is calculated in (33)–(39). The DL-based detector model was obtained based on the TensorFlow library so, for a fair comparison, the number of multiplications and summations will be counted following the DNN used model and the DL layers size. Table 3 shows the receiver’s complexity in terms of the TNCO for the proposed scheme in comparison with the conventional SMTs. As shown in Table 3, the proposed DLSS-FGSM significantly reduces the receiver’s detector complexity compared to SM, GSM, FGSM, QSM, and EFGSM. While, the GCIM and EFGSM slightly outperform the proposed scheme in the receiver’s computation, but they have a poor ABER performance over the underwater acoustic channel. Furthermore, Table 3 shows the high competition complexity of the DLSS-FGSM scheme based on the ML-based detector which restricted its use in underwater communication.

## 6. Conclusions

In this paper, a new spatial modulation technique named SS-FGSM is proposed for the underwater acoustic channel. The proposed SS-FGSM scheme uses two axes (antenna index and spreading codes index) for the spatial domain to convey additional information bits and increase the achievable data rate. Unlike the traditional SMTs schemes, the proposed scheme uses three dimensions for data transmission, the habitual M-ary signal constellation, antenna index, and spreading code index. Two axes of the spatial domain have been used for conveying data transmission leading to reduce the physical bits transmitted over the channel and increase the SE and EE of the communication system. Additionally, the power consumption has been saved by transmitting only a few bits physically over the channel and most bits are transmitted based on indices of either the transmit antennas or the spreading codes. For the receiver’s complexity point of view, the proposed scheme is evaluated based on two types of the detector; ML-based detector and DL-based detector. Due to the high computational complexity of the ML decoder, this paper provides a deep learning-based detector to be used in underwater communication to avoid wasting additional computational power. The general ABER formula for MLSS-FGSM wasderived to evaluate the proposed scheme performance, and the simulation analysis confirmed the corresponding mathematical expressions used in the ABER analysis. Furthermore, the SE, EE, and receiver’s complexity of the proposed scheme is analyzed and compared with different SMTs. The proposed scheme has been evaluated by using measured underwater acoustic channels conducted from one sea-going experiment. In particular, the proposed scheme outperforms the conventional schemes in terms of energy and spectrum efficiency, as well as in terms of ABER performance. In our future work, real experimental tests for the proposed scheme is going to be conducted.

## Figures and Tables

**Table 1:** Notations.

Description	Notation	Description	Notation	Description	Notation	Description	Notation
Number of transmit antennas	$N_{t}$	Walsh codes	W	Number of available spreading code	N	Imaginary part of the block of bits	$d_{Q}$
Number of receive antennas	$N_{r}$	Number of code chips	L	Energy required for transmitted bit	$E_{b}$	Weights of layers	${𝓅}_{w}$
The achieved data rate	R	Chip period	$T_{c}$	Doppler scaling factor	β	The transmitted signal	$x\left(t\right)$
Modulation order	M	Pulse shaping filter	$p\left(t\right)$	Channel delays	τ	Block of bits	B
Number of multipath	μ	Carrier frequency	$f_{o}$	Modulated symbol	$s_{𝒾}$	Learning rate	α
Number of active antennas	$N_{u}$	Biases of the layers	${𝒻}_{w}$	Additive white Gaussian noise	n	Real part of the block of bits	$d_{I}$
Index of the active antenna	q	Probability associated with erroneous spreading code	$F_{c}$	Channel time-varying path amplitudes	A	Probability associated with the modulated bits	$F_{m}$
Real part of modulated symbol	$a_{𝒾}$	Length of the first hidden later	Q				

**Table 2:** Example of SS-FGSM procedure.

Transmitted Bits			Antenna Combination
Data Bits	Codes Bits	Antennas Bits	
$d_{M-ary,𝒾}^{T}$	$d_{I,𝒾}^{T}d_{Q,𝒾}^{T}$	000	$T_{x1}$
$d_{M-ary,𝒾}^{T}$	$d_{I,𝒾}^{T}d_{Q,𝒾}^{T}$	001	$T_{x2}$
$d_{M-ary,𝒾}^{T}$	$d_{I,𝒾}^{T}d_{Q,𝒾}^{T}$	010	$T_{x3}$
$d_{M-ary,𝒾}^{T}$	$d_{I,𝒾}^{T}d_{Q,𝒾}^{T}$	011	$T_{x4}$
$d_{M-ary,𝒾}^{T}$	$d_{I,𝒾}^{T}d_{Q,𝒾}^{T}$	100	$T_{x1}T_{x2}$
$d_{M-ary,𝒾}^{T}$	$d_{I,𝒾}^{T}d_{Q,𝒾}^{T}$	101	$T_{x1}T_{x3}$
$d_{M-ary,𝒾}^{T}$	$d_{I,𝒾}^{T}d_{Q,𝒾}^{T}$	110	$T_{x1}T_{x4}$
$d_{M-ary,𝒾}^{T}$	$d_{I,𝒾}^{T}d_{Q,𝒾}^{T}$	111	$T_{x2}T_{x4}$

**Table 3:** Performance comparison of Complexity.

SM	32,768
GSM	65,536
QSM	32,768
FGSM	65,536
EFGSM	1280
GCIM	352
MLSS-FGSM	262,144
DLSS-FGSM	5376
